# Implementing ACL Injury Prevention in Daily Sports Practice—It’s Not Just the Program: Let’s Build Together, Involve the Context, and Improve the Content

**DOI:** 10.1007/s40279-021-01560-4

**Published:** 2021-09-17

**Authors:** Anne Benjaminse, Evert Verhagen

**Affiliations:** 1 Department of Human Movement Sciences, University of Groningen, UMC Groningen, Antonius Deusinglaan 1, 9713 AV Groningen, The Netherlands; 2 Department of Public and Occupational Health, Amsterdam Collaboration on Health and Safety in Sports, Amsterdam Movement Sciences, Amsterdam UMC, University Medical Centres-Vrije Universiteit Amsterdam, Amsterdam, The Netherlands; 3 School of Sport Studies, Hanze University Groningen, Groningen, The Netherlands

## Abstract

Although the benefits of current anterior cruciate ligament (ACL) injury prevention programmes have been demonstrated in efficacy studies, they, unfortunately, have had limited public health impact to date. For example, the incidence of ACL injuries continues to rise in adolescent athletes. Raising awareness and educating coaches and athletes is not enough to facilitate the widespread, sustained use of these programmes in the real-world setting. Considering the profound burden of ACL injuries, it is necessary to continue to improve the current ACL injury prevention programmes through co-creation. First, the uptake of the programmes should be optimized by a better appreciation and understanding of the individual, socio-cultural and environmental *context* (i.e., community). Second, the *content* of the programmes should be optimized to better reflect the demands of the sport by creating more ownership and increasing motivation (incorporating challenging, sport-specific and fun elements) with the end-users. In addition, implicit motor learning, random practice and differential learning are concepts that should be integrated when practising to obtain the most optimal results when learning or finetuning skills.

## Key Points

**Table:** 

Considering the profound burden of ACL injuries, it is necessary to continue to improve the current ACL injury prevention programs through co-creation with the end-users.
The context of the player and the demands of the sport (content) should be included when designing ACL injury prevention programs.
Motor learning methods that induce high practice variability to guide a player’s search for his/her optimal movement solution are promising and warranted.

## Introduction

Engaging in sport is one way of being physically active, and sports participation has a great positive influence on the level of health-enhancing physical activity and mental well-being [1, 2]. Despite the health benefits of sports activities, sports injury and fear of (re-)injury are real barriers to sport participation. The risk of sports injuries substantially increases during youth, peaking in the 15- to 19-years age group [3]. Injuries may affect the athlete’s career and their daily-life activities. One reason for non-participation in and drop-out from sports is sports-related injuries [4, 5]. Knee and ankle injuries in particular contribute to this problem [6]. One severe knee injury with increasing incidence is the anterior cruciate ligament (ACL) tear, which, in many cases, requires surgical intervention [6]. Per year, over two million ACL injuries occur worldwide. The majority of these injuries are observed in paediatric and adolescent athletes [6–10], with a higher incidence in girls compared to boys, especially at younger ages [11–13]. These ACL injuries lead to the longest withdrawal time from youth sports. Only 44% of these young athletes return to their pre-injury level of sports, and up to 23% sustain a re-injury, with many of them dropping out of sports entirely [14]. An ACL rupture is a devastating injury for a soccer player, resulting in the longest layoff times [15] and a reduced career length [16]. This change in life immensely affects both physical and psychosocial well-being in the short and long term. An ACL injury is associated with an increased risk of physical health issues: decreased sport participation [14], decreased physical activity [17] and increased body mass index (BMI) [18], high risk of subsequent knee injuries [19], and early-onset knee osteoarthritis [20]. The seriousness and severity of the ACL injury are reflected in the intensive, extended physical rehabilitation process that is essential for an optimal outcome [15]. The psychosocial impact of such a long recovery process can be particularly devastating. A lack of mobility may result in post-traumatic stress, depression, fear of re-injury, social isolation [21, 22], mood disturbances, anger [23], decreased locus of control, lowered self-efficacy or loss of self-worth as a result of not being able to perform pre-injury state functions [24, 25]. Thus, many health benefits are lost due to injuries, negatively affecting a healthy lifestyle and public health. Health gains associated with sports and physical activities need to be optimised by ensuring appropriate and efficacious preventative interventions are in place. Much effort has been invested on preventing ACL injuries, for example the potential reduction of (non-contact) ACL injuries can be up to 67% in females in controlled settings [26]. However, with regard to effectiveness, ACL injury rates have not decreased over recent decades in the real world [27]. To bridge this gap, we should bring context to the exercises. On the one hand, we can do this by developing ACL injury-prevention programmes and stakeholders as a shared responsibility. On the other hand, we should look more at the demands of the sports and how to optimally teach these exercises (content).

## Why Aren't Efficacious Anterior Cruciate Ligament (ACL) Injury-Prevention Programmes Adopted?

Many ACL injury-prevention programmes provide coaches and sports teams with warm-up exercises [28–30]. Even though showing promising efficacy results, unfortunately the programmes are not well implemented in real-world settings [31–33], often because they do not match specific contexts [34, 35]. Often, coaches experience these exercises as being too static and not sport-specific, insufficiently supporting their performance goals, insufficiently challenging the athlete, and not being tailored to the individual athlete [31]. Implementation in the real-world setting therefore remains a major challenge [36]. Because of these barriers, the exercises are not experienced as being attractive and applicable [31, 37], and only a few coaches are actually using the exercises, leading to less optimal effectiveness than was expected from controlled research [28–30]. Lack of uptake and maintenance of such programmes are thus of ongoing concern, and adoption, fidelity and maintenance are challenging [38]. Implementation efforts focus on singular, static exercises for the end-users (coaches, athletes) without considering individual contextual situations leading to tailor-made solutions [39]. For the durability of these ACL injury-prevention programmes and for them to be effective in the real world, it is necessary to design them taking the real world into account in terms of better considering both the context and the programmes' content.

## Now What?

### Context

It is time to listen to and recognise coaches’ needs, barriers, knowledge and ideas, and promote a co-learning and empowering process between the scientific and practical fields [39]. Implementation needs to be made sustainable, and exercises must be created jointly by theoretical and practical experts. The sports medicine research field still approaches solutions with a linear *tame problem* approach (i.e., implying that one optimal solution exists) [34, 40]. However, preventing sports injuries poses a so-called *wicked problem* (i.e., implying multiple solutions exist) [41] and is not amenable to top-down general solutions. This implies that multiple optimal solutions exist, depending on the setting, such as level and type of sports, club culture and coach’s knowledge. Suppose we consider sports injuries a wicked, complex phenomenon [42, 43] and ACL injury prevention as a complex system. In that case, it requires multiple stakeholders working together and sharing the responsibility to prevent injuries. Systems thinking helps us understand how the elements related to ACL injury and its prevention are under the control of different stakeholders [44]. These different stakeholders (e.g., coaches, sports physiotherapists, parents, club administrators, and regional and national federation administrators) relate to the athlete at different levels, that is, individual, socio-cultural and environmental levels [45]. Therefore, to develop more comprehensive ACL injury-prevention strategies, ideally all stakeholders should be involved from the beginning by acknowledging how their roles potentially impact injury and its prevention [44, 46]. If end-users are not engaged initially, their knowledge and experience are not implemented to their full potential, and exercises are often modified because they do not match their context [31, 37]. Delivering programme content supported by a context-specific and evidence-informed implementation plan leads to greater implementation activity, which is important for injury reduction [31].

ACL injury-prevention efforts need to be built around coaching behaviours to be effective. If not, this will lead to the development of context-free preventive solutions. ACL injury prevention should focus on ‘what works for whom, when, where, and why’ [47]. Consequently, there is a need to know and understand more about the behavioural aspects related to ACL injury occurrence. Thus, contextual factors should be considered early on in the development of ACL injury prevention intervention and not just in the implementation phase. One important example of doing so is considering the coach to be central in their context (i.e., human-centred design) and engaging them to understand their preferences [48]. This user-centredness and design thinking prioritises a deep empathy for the desires, needs and challenges of end-users to fully understand a problem and develop more comprehensive and effective solutions [49]. Design thinking promotes opposing ideas and debate (divergent), and aims to uncover what is important to consumers in their everyday lives. Therefore, the co-creation process with coaches is a non-linear process that requires a comprehensive understanding of underlying problems and follows theoretical demands. This process starts with the coaches, involving them in the creation process, for example through pilots and consolidations (i.e., cyclical and iterative intervention development and evaluation process) [39]. It should include mapping of current barriers and beliefs, their alignment, and development of content through discussion, consensus meetings and practical validations, all involving the end-users. The process ends with interventions that are tailor-made to suit context-specific needs [50]. A focus on implementation is, therefore, critical to influence behaviour change and give coaches ownership. Engaging coaches to understand their knowledge, ideas, wishes, needs and preferences is crucial for reaching sustainable, evidence-informed injury-prevention practice [44]. The exact process of co-creation depends on the context. For example, how in-depth the input from coaches along the process will be, will depend on availability, level of sport and education, experience, etc. The golden rule is to design a programme with their input and with their agreement. When developing ACL injury-prevention programmes, the focus should be set on the necessity of sustainable implementation through applicability in the real-world setting.

### Content

Exercises should ideally be fun, sport-specific, challenging and individualised. This will enhance both the athlete’s and the coach’s satisfaction with the programme [31], and improves motor learning [51]. A non-contact ACL injury results from a failure of the system to effectively self-organise movements within the quickly changing constraints present. Current ACL injury-prevention exercises do not reflect the neurocognitive and physical demands of ball-team sports [52] as learning traditional, closed-skill anticipated exercises could not account for this continuous changing environment during a game in which injuries typically occur and where the athletes have to move and perform optimally. Therefore, the ability to perform pre-planned movement tasks is poor preparation for the dynamic, responsive and unplanned motor tasks that occur in sports. It is time now to take the complex and unpredictable sports environment into account when learning adaptive motor skills. Situational awareness, arousal and attentional resources of the individual affect the complex integration of vestibular, visual and somatosensory information needed for neuromuscular control [53]. The impact of the quickly changing environment, and the athlete’s ability to perceive and interpret this and select an appropriate motor response can thus not be ignored when ACL injury reduction is the goal. In other words, neurocognitive skills such as visual attention, processing speed and dual tasking (lower-order), and inhibition, working memory and cognitive flexibility (core) impact lower extremity biomechanics [54]. Exercises should thus also account for practising these neurocognitive skills in combination with the motor task [51, 55].

It is good to search for the best practice conditions that engage the learner in an effortful learning process and have been shown to enhance retention and transfer [56]. For retention (i.e., long-term effectiveness) and transfer of skills to occur, incorporating the key concepts for practice outlined below may be more effective in preventing injuries [57–59]. First, *implicit motor learning* (such as, among others, analogy learning, the external focus of attention) and *motivation*, stimulated by (a) self-controlled learning (enhancing feelings of autonomy) and (b) enhanced expectancies (enhancing feelings of competence and self-efficacy) [60], should be considered.

#### Optimal Motor Learning—Implicit Learning

Implicit learning methods aim to minimise declarative (explicit) knowledge about movement execution during learning. For this purpose, implicit learning can be induced by providing external focus instructions or analogies rather than explicit instructions during motor skill acquisition. Implicit learning reduces the reliance on the working memory and promotes more of an automatic process [61]. It is for this reason that it can be more effective in more complex tasks. Competitive sports can be psychologically demanding, and decision-making accuracy deteriorates in athletes under pressure, involving increased task complexity [62]. Implicit motor learning has been shown to be more sustainable in situations with physical [63–65] or mental pressure [66, 67], which is very relevant to the sports context. For example, applying implicit motor learning when practising optimising agility movements could be asking the athlete to ‘pretend your knees are headlights and point them towards the new direction’ (analogy instruction) [68] or ‘when turning, push yourself as forcefully as possible from the ground’ (external focus instruction) [69] to enhance movement form in the frontal and sagittal planes, respectively.

#### Optimal Motor Learning—Motivation

Learning is a problem-solving process, and the athlete's involvement during practice to search for his or her own (movement) solutions enhances learning. Having some choice appeals to one of the basic psychological needs of human beings and enhances intrinsic motivation [59]. Having some choice can stimulate beliefs in one's capabilities (competence) and enhance feelings of self-efficacy, for example choosing the variety of the exercises the athlete thinks s/he can do best or that challenged him or her most. Experiencing the exercises to be challenging and sport-specific makes them meaningful and will give a motivational boost. In summary, it has been demonstrated that motivation is crucial to improve motor skill learning [60]. Human motivation is dependent on (the perception of) one’s actions having effects on the environment [70]. Positive expectations for the near future (feelings of 'yes I can do this’), as well as perceptions of autonomy, are intrinsic to motivation [60]. Circumstances that enhance learners’ expectations and confidence for future performance success enhance movement automaticity and improve motor learning [71]. Even if the effects of one’s actions are trivial, intrinsic motivation is enhanced if the person has control over those effects [70]. Conditions that provide an opportunity for choice may be motivating because they indicate control over upcoming events. Therefore, it is advised that coaches try to stimulate the players’ enjoyment, needs satisfaction, or sense of challenge or curiosity during the activity [59]. For example, when practising an agility manoeuvre, the coach can give the players a choice about which variation of the task to practice (e.g., practising the task with different materials or practising the task at different difficulty levels). Another way to provide feelings of autonomy is by asking questions: “How can you make this exercise more challenging for yourself?” or “Let me know when you're ready to move to the next level of this exercise”. Also, the use of non-controlling language means the avoidance of words such as ‘should,’ ‘must’ and ‘have to’ to convey a sense of choice or flexibility [72].

#### Training Design

While performing a movement, team sports players have to quickly visually perceive their action opportunities and those of opponents and teammates. These continuous actions are performed under time pressure as movement possibilities emerge and disappear. Therefore, a non-contact ACL injury is the result of a series of self-organised movements that emerge from the interaction with quickly changing constraints. This means an injurious movement is not produced by an isolated player but emerges from a dynamically varying association between the player’s characteristics, the stimulus-rich environment and the desired actions [73–76]. Considering this ecological dynamics approach, presenting players with varying game-like variables so that the elicited movement is more reflective of the movements in injury scenarios may be beneficial. Random practice or differential learning may be options to do this.

*Random practice* (i.e. practising multiple skills in random order with high contextual interference) while adding various constraints should be considered for improving motor learning [51, 58]. The absence of the consecutive repetition of a given skill during a random execution sequence leads to poorer direct performance than does experiencing a sequence in blocks [77]. However, the poor direct performance levels of those who practice in a random order mask the greater psychophysiological demands of subcortical structures that this type of condition requires. This increased participation during the practice by brain regions involved in motor skill planning and execution [78], which is reflected by a higher activation level and cortical excitability, is a critical factor in learning consolidation [57]. The opposite effect is observed in retention testing, during which random practice leads to decreased activation levels in the indicated regions [57], leading to greater automaticity of movement. The variable practice involves performing variations of the task or completely different tasks throughout a training session [57]. This means, for example, mixing unanticipated deceleration and cutting in different directions, jumping and single-leg landing on the left and right leg, with and without the ball, from different angles, at different speeds, etc.

Lastly, *differential learning* can be considered to enhance motor learning. Differential learning means that players randomly perform various movement patterns when practising a skill (rather than only practising the supposedly ‘correct’ movement form). They are stimulated to engage in a self-organised learning process [79]. This can be done by adding a task or environmental constraints that ‘force’ them to execute the same task differently. For example, passing a football or tennis ball while cutting; cutting while juggling with a tennis ball; having teammates throw or pass balls at the player; having the opponent defend while doing an agility parkour; performing some single leg hops, a turn or a funny jump before cutting; performing cuts on sand, asphalt, within a limited space, etc. The coach should be creative and make it fun for the players. The purpose is to develop control over the body's many degrees of freedom, and have adaptable movement solutions available, rather than training for the ‘ideal' movement technique, limiting movement solutions for variable tasks.

Future research is needed to evaluate the effectiveness of such proposed elements in ACL injury-prevention programmes. However, motor learning methods that induce great practice variability to guide a player’s search for his/her optimal movement solution are promising and warranted [80].

## Conclusion

We do not have the programme; we are not there yet. Including coaches and co-creating (context) and adopting motor learning principles (content) may help improve ACL injury-prevention implementation. For programmes to be more effective, they should also better reflect the sporting demands. If people do not use the available programmes, the programmes are not good enough. In addition, future efforts should focus on the enhancement of current programmes as well as successful implementation through leadership, management and education.
